# Synthesized HMGB1 peptide attenuates liver inflammation and suppresses fibrosis in mice

**DOI:** 10.1186/s41232-021-00177-4

**Published:** 2021-09-27

**Authors:** Shunsuke Nojiri, Atsunori Tsuchiya, Kazuki Natsui, Suguru Takeuchi, Takayuki Watanabe, Yuichi Kojima, Yusuke Watanabe, Hiroteru Kamimura, Masahiro Ogawa, Satoko Motegi, Takahiro Iwasawa, Takeki Sato, Masaru Kumagai, Yui Ishii, Tomomi Kitayama, Yu-Tung Li, Yuya Ouchi, Takashi Shimbo, Masaaki Takamura, Katsuto Tamai, Shuji Terai

**Affiliations:** 1 Division of Gastroenterology and Hepatology, Graduate School of Medical and Dental Sciences, Niigata University, 1-757, Asahimachi-dori, Chuo-ku, Niigata, 951-8510 Japan; 2 Department of Stem Cell Therapy Science, Graduate School of Medicine, Osaka University, 2-2, Yamadaoka, Suita, Osaka, 565-0871 Japan; 3 StemRIM Inc., Saito Bio-Incubator 3F 7-7-15, Saito-Asagi, Ibaraki City, Osaka, 567-0085 Japan; 4 StemRIM Institute of Regeneration-Inducing Medicine, Osaka University, 2-8, Yamadaoka, Suita, Osaka, 565-0871 Japan

**Keywords:** HMGB1, Peptide, Liver, Cirrhosis, Fibrosis, Mesenchymal stem cell, Macrophage, Mouse, Scar-associated macrophage, Single-cell transcriptome analysis

## Abstract

The liver has a high regenerative ability and can induce spontaneous regression of fibrosis when early liver damage occurs; however, these abilities are lost when chronic liver damage results in decompensated cirrhosis. Cell therapies, such as mesenchymal stem cell (MSC) and macrophage therapies, have attracted attention as potential strategies for mitigating liver fibrosis. Here, we evaluated the therapeutic effects of HMGB1 peptide synthesized from box A of high mobility group box 1 protein. Liver damage and fibrosis were evaluated using a carbon tetrachloride (CCl_4_)-induced cirrhosis mouse model. The effects of HMGB1 peptide against immune cells were evaluated by single-cell RNA-seq using liver tissues, and those against monocytes/macrophages were further evaluated by in vitro analyses. Administration of HMGB1 peptide did not elicit a rapid response within 36 h, but attenuated liver damage after 1 week and suppressed fibrosis after 2 weeks. Fibrosis regression developed over time, despite continuous liver damage, suggesting that administration of this peptide could induce fibrolysis. In vitro analyses could not confirm a direct effect of HMGB1 peptide against monocyte/macrophages. However, macrophages were the most affected immune cells in the liver, and the number of scar-associated macrophages (*Trem2*+*Cd9*+ cells) with anti-inflammatory markers increased in the liver following HMGB1 treatment, suggesting that indirect effects of monocytes/macrophages were important for therapeutic efficacy. Overall, we established a new concept for cell-free therapy using HMGB1 peptide for cirrhosis through the induction of anti-inflammatory macrophages.

**Supplementary Information:**

The online version contains supplementary material available at 10.1186/s41232-021-00177-4.

## Introduction

The liver has a high regenerative ability and can induce spontaneous regression of fibrosis when early liver damage occurs resolving chronic liver diseases. However, when chronic liver damage continues for a long time, decompensated cirrhosis occurs, and the ability of the liver to regenerate and alleviate fibrosis is lost; in such cases, only liver transplantation can cure patients. Thus, therapy for inducing endogenous regeneration ability and fibrosis regression is essential [1, 2].

These endogenous systems are primarily induced by immune cells, particularly macrophages. Duffield et al. reported that during liver damage and subsequent scarring, macrophages can have two opposing roles, exhibiting opposite polarity and playing critical roles in both the injury and recovery phases of inflammatory scarring [3]. Additionally, Thomas et al. reported that regression of fibrosis could be achieved by injecting bone marrow-derived macrophages through the portal vein [4]. Based on these results, Forbes et al. performed a phase I study using an injection of autologous cultured macrophages for cirrhosis; the approach was shown to be safe, and researchers have moved to phase II studies of this treatment [5].

Another therapy that can induce these endogenous systems is that using mesenchymal stem cells (MSCs). MSCs are stem cells that can be differentiated into osteocytes, chondrocytes, and adipocytes and can be harvested from bone marrow and medical waste, such as adipose tissue, umbilical cord tissue, and dental pulp [2]. In a mouse study of MSCs, Watanabe et al. reported that MSCs and macrophages synergistically regress liver fibrosis and improve liver regeneration. Observation using two-photon excitation microscopy revealed that most MSCs migrate to the lung and function as “conducting” cells to direct effector macrophages to the damaged area of the liver [6]. Many other reports have also described the roles of macrophages in MSC therapy [7]. Accordingly, induction of anti-inflammatory or scar-resolving macrophages is fundamental for the treatment of cirrhosis, and substances or drugs inducing these macrophages may have applications in future cell-free therapies [1, 2].

High mobility group box 1 (HMGB1) is a nonhistone nuclear protein that regulates chromatin structure remodeling as a molecular chaperone in the chromatin DNA-protein complex [8]. This HMGB1 has two opposite roles, i.e., supporting tissue repair [9] and inducing inflammation [10–12], carried out via lesion boxes A and B. In injured or infected tissues, HMGB1 is actively secreted by macrophages and dendritic cells or passively released from necrotic cells, and box B of HMGB1 induces tissue remodeling by activating inflammatory reactions, i.e., macrophage and neutrophil infiltration, via binding to Toll-like receptors (TLRs) and/or receptor for advanced glycation end-products on their surfaces [13]. In contrast, box A of HMGB1 inhibits TLR signals and blocks the spread of inflammation to the surrounding tissues [11, 14]. However, the roles of this molecule in liver fibrosis have not yet been reported.

In this study, we analyzed the therapeutic effects of a peptide synthesized from box A of HMGB1 (called HMGB1 peptide) on macrophage function and liver damage in a carbon tetrachloride (CCl_4_)-induced cirrhosis mouse model.

## Methods

### Mice

C57BL/6 male mice were purchased from Charles River (Yokohama, Japan). Animals were housed in a specific pathogen-free environment and kept under standard conditions with a 12-h day/night cycle and access to food and water ad libitum. All animal experiments were conducted in compliance with institutional regulations, ARRIVE guidelines, and the study protocols were approved by the Institutional Animal Care and Committee of Niigata University and Osaka University.

### HMGB1 peptide

HMGB1 peptide (lot. no. AEF93//449-020) was provided by StemRim Inc. (Osaka, Japan) [15]. Before injection, HMGB1 peptide was dissolved in normal saline (NS, concentration: 1 mg/mL; Otsuka Pharmaceutical Co., Ltd., Tokyo, Japan).

### CCl_4_-induced cirrhosis mouse model

To establish the CCl_4_-induced cirrhosis mouse model, male mice (10 weeks old) were intraperitoneally injected with 1.0 mL/kg CCl4 (FUJIFILM Wako Pure Chemical Industries Ltd., Osaka, Japan) dissolved in corn oil (FUJIFILM Wako Pure Chemical Industries Ltd.) at a 1:10 volumetric ratio twice weekly until sacrifice for analysis of cirrhosis. Eight weeks after CCl_4_ injection, NS (control; 5 μL/g) or HMGB1 peptide (5 μg/g) was administered via the tail vein twice a week for 1, 2, or 4 weeks. During the HMGB1 peptide injection period, CCl_4_ was continually administered. Three days after the final HMGB1 peptide or CCl_4_ administration, serum and fibrosis analysis were performed. In the one-time CCl_4_ injection experiment, HMGB1 peptide (5 μg/g) was injected two times (3 days prior to CCl_4_ injection [same dose as mentioned above] and same day of CCl_4_ injection). Thirty-six hours after CCl_4_ injection, serum and tissues were obtained.

### Macrophage culture and assay

Bone-marrow cells collected from the femurs of 10-week-old male mice were cultured at 37 °C in the presence of 5% CO_2_ in ultra-low attachment flasks (Corning, Corning, NY, USA) and medium (Dulbecco’s modified Eagle’s medium/F12; Thermo Fisher Scientific) containing 20 ng/mL macrophage colony-stimulating factor-1 (Peprotech Inc., Rocky Hill, NJ, USA); the medium was changed twice weekly, as described previously [6]. After 7 days, the macrophages were harvested. The harvested macrophages were seeded in 6-well ultra-low attachment dishes (Corning) at a density of 5 × 10^6^ cells/well. Then, 0.1 mg HMGB1 peptide (concentration: 1 mg/mL), 0.01 mg HMGB1 peptide (concentration: 0.1 mg/mL, low-dose group), or NS (control group) was added to the cultured macrophages. After 48 h, macrophages were harvested, and mRNA expression levels of genes encoding pro-inflammatory factors (e.g., interleukin-6 [*Il6*], tumor necrosis factor [*Tnfa*], monocyte chemotactic protein-1 [*Mcp1*], and inducible nitric oxide synthase [*Inos*]) and anti-inflammatory factors (e.g., *Il-10,* chitinase 3-like 3 [*Ym1*], found in inflammatory zone protein [*Fizz1*], and *Cd206*) were evaluated using real-time polymerase chain reaction (PCR).

### Real-time PCR

Total RNA was obtained using an RNeasy kit (Qiagen, Venlo, Netherlands) and was reverse transcribed using a QuantiTect reverse transcription kit (Qiagen). Gene expression analysis was performed using prevalidated QuantiTect primers (Supplemental Table 1) with QuantiTect SYBR reagent (Qiagen). Real-time PCR was conducted with a Step One Plus Real-time PCR System (Applied Biosystems, Foster City, CA, USA). The results were obtained from five separate samples. The gene encoding glyceraldehyde 3-phosphate dehydrogenase was used as an internal control. The fold change in relative gene expression from the control was calculated using the ΔΔCt method.

### Serum analyses

Arterial blood samples were obtained from the hearts of mice at 1, 2, and 4 weeks after starting HMGB1 peptide injection. Serum alanine aminotransferase (ALT), aspartate transaminase (AST), total bilirubin (Bil), and albumin (ALB) concentrations were determined by Oriental Yeast Co., Ltd., Nagahama LSL (Nagahama, Japan).

### Sirius Red staining

To quantify fibrosis, liver tissues were collected at 1, 2, and 4 weeks after starting HMGB1 peptide injection. Tissues were fixed with 10% formalin, cut into 3-μm-thick sections, and stained with Sirius Red. Photographs were captured from each section randomly (10 fields/mouse) using a BZ-9000 microscope (Keyence, Osaka, Japan), and quantitative analysis of the fibrotic area was performed using ImageJ software (version 1.6.0 20, National Institutes of Health, Bethesda, MD, USA).

### Hydroxyproline assay

The levels of hydroxyproline, a representative collagen component, were determined in the liver cirrhosis mouse model at 1, 2, and 4 weeks after starting HMGB1 peptide injection. Liver samples (20 mg) were homogenized and subjected to QuickZyme Hydroxyproline Assays (QuickZyme Bioscience, Zernikedreef, the Netherlands) according to the manufacturer’s protocol. Samples were extracted, and absorbance was measured at 570 nm. Data were expressed as the amount of hydroxyproline per 1-mg liver tissue.

### Immunohistochemistry

For staining the liver tissue, 10% formalin-fixed tissue was sliced into 4-μm-thick sections. Immunohistochemistry for F4/80 (ab111101; rabbit monoclonal to F4/80; Abcam, Cambridge, UK) was performed as follows. The dewaxed tissues were subjected to antigen retrieval in EDTA buffer (pH 8.0) for 20 min using a microwave. The primary antibody was applied overnight in an antibody diluent reagent solution (Thermo Fisher Scientific). The secondary antibody reaction was performed using the Vecstain ABC kit (Vector Laboratories, Burlingame, CA, USA). The sections were stained by reaction with DAB TRIS tablets (Muto Pure Chemicals, Tokyo, Japan). Photographs were captured from each section randomly (20 fields/mouse) using an OLYMPUS CX33 microscope (OLYMPUS, Tokyo, Japan).

### Single-cell transcriptome analysis

To obtain single-cell suspensions from livers for single-cell RNA-sequencing, mouse livers were perfused and enzymatically digested using a liver dissociation kit (Miltenyi Biotec). Debris was removed with 70- and 40-μm filters (Corning). Nonparenchymal cells were enriched by centrifugation using 25% Percoll (GE Healthcare, Chicago, USA). The resulting cells were stained with allophycocyanin-anti-CD45 antibodies (BioLegend, San Diego, USA). Live, CD45-positive cells were sorted into 384-well plates (Eppendorf, Hamburg, Deutschland) using a BD FACSAria III instrument (Becton Dickinson; 100 μm chip) in single-cell purity mode (1 cell/well). Single-cell RNA-seq libraries were constructed based on a previous report [16] with some modifications. Briefly, cells were lysed, and RNA was reverse transcribed using barcoded oligo dT primers (5′-ACGACGCTCTTCCGATCT[Barcode]NNNNNNNNTTTTTTTTTTTTTTTTTTTTTTTTTTTTTTVN-3′, where “N” is any base and “V” is either “A”, “C” or “G”; IDT). Resulting cDNA was amplified using an Accel-NGS 1S Plus DNA Library Kit (Swift Biosciences, Ann Arbor, MI, USA) and KAPA HiFi HotStart ReadyMix (KAPA Biosystems, Boston, MA, USA) with i5 primer (5′-AATGATACGGCGACCACCGAGATCTACAC[i5]ACACTCTTTCCCTACACGACGCTCTTCCGATCT-3′; IDT) and D7 primer (5′-CAAGCAGAAGACGGCATACGAGATCGAGTAATGTGACTGGAGTTCAGACGTGTGCTCTTCCGATC-3′; IDT). Amplified cDNA was further treated with Nextera TD buffer (Illumina, San Diego, CA, USA) and 5 μL Amplicon Tagment enzyme (Illumina) and then amplified with i5 primer and P7 primer (5′-CAAGCAGAAGACGGCATACGAGAT[i7]GTCTCGTGGGCTCGG-3′). Libraries were purified and sequenced on a NextSeq500 platform under the following conditions: 20 (read 1) + 8 (i7) + 8 (i5) + 51 (read 2) bases. The sequencing libraries were sequenced on a NextSeq500 platform (Illumina). The read length was set to 20 (read 1) + 8 (i7) + 8 (i5) + 51 (read 2) bases. Sequencing outputs were demultiplexed using bcl2fastq2 (https://jp.support.illumina.com/sequencing/sequencing_software/bcl2fastq-conversion-software.html). Fastq files were aligned to *Mus musculus* GRCm38 reference using STAR aligner (2.7.1a; https://github.com/alexdobin/STAR). STARsolo outputs were filtered using the numbers of reads, transcripts, and genes and the percentages of mitochondrial genes and mapped reads for each cell. Downstream analysis was performed using Monocle3 R package [17–19]. To isolate macrophages for functional analyses after HMGB1 treatment, cell clusters positive for the hepatic macrophage markers epidermal growth factor-like module-containing, mucin-like hormone receptor 1 (EMR1), CD68, and C-type lectin domain family 4 member F (CLEC4F) were reclustered twice to remove nonmacrophage cells. Macrophages were aggregated by clustering, and gene module analysis was performed on genes showing uneven distributions on the Uniform Manifold Approximation and Projection (UMAP; Moran I greater than 0 and q-value below 0.01). The expression of gene modules in each macrophage cluster was scaled to *z*-scores, and modules showing a difference greater than 2 standard deviations (SDs) to the mean *z*-score of all macrophage clusters were pooled for Gene Ontology biological process analysis with a *q*-value threshold of 0.01. To compare gene expression in general, cells were aggregated into groups by treatment or cluster, and expression in each group was normalized to mean expression.

### Statistical analyses

Statistical analysis was performed by using GraphPad Prism8 software (GraphPad Software Inc., La Jolla, CA, USA), R (Free software by The R Foundation, Vienna, Austria), and Microsoft Excel (Microsoft, Washington, DC, USA). Data are presented as means ± SDs. The results were assessed using Welch’s *t*-test. Differences between groups were analyzed by Welch’s one-way analysis of variance. Differences with *p* values of less than 0.05 were considered significant.

## Results

### Injection of HMGB1 peptide attenuated liver damage and promoted the regression of fibrosis in model mice with CCl_4_-induced liver damage

**Fig. 1:**
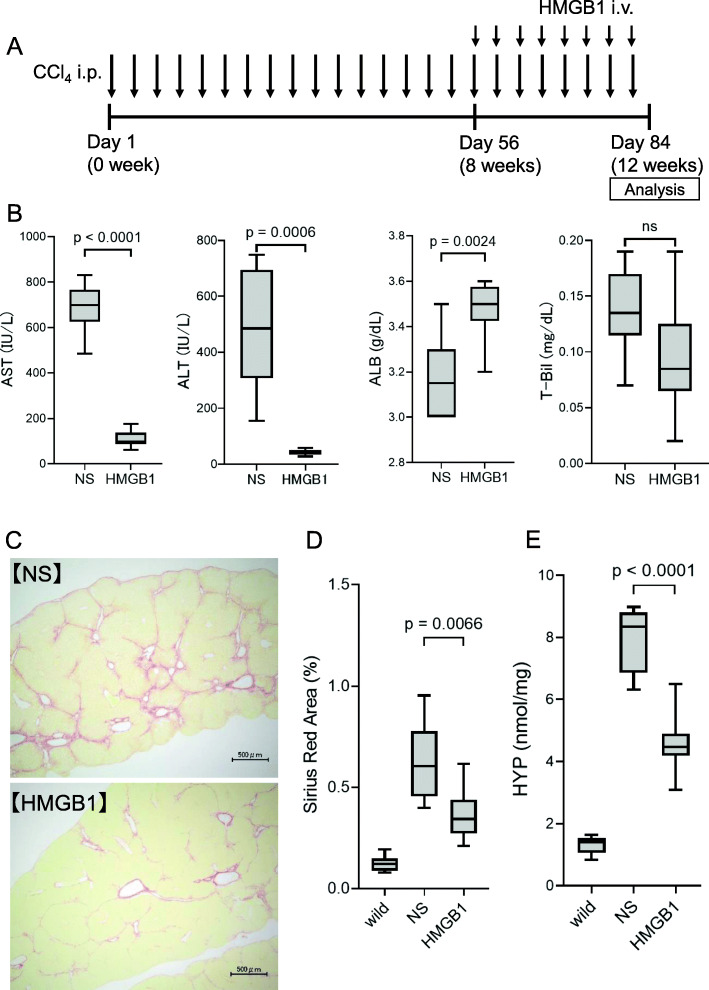
Therapeutic effects of HMGB1 peptide (4 weeks of treatment) in the CCl_4_-induced mouse cirrhosis model. **A** Schematic of this experiment. **B** Serum levels of aspartate transaminase (AST), alanine transaminase (ALT), albumin (ALB), and total bilirubin (T-Bil). **C** Sirius Red staining of liver tissues. Scale bar = 500 μm. **D** The frequency of Sirius Red staining. **E** Quantification of hydroxyproline (HYP). NS, normal saline, *n* = 8 in each group. Data are presented as means ± standard deviations. ns, not significant. wild, basic data of from undamaged normal liver at the same age

To evaluate the therapeutic effects of HMGB1 peptide, HMGB1 peptide was injected into mice twice a week for 4 weeks using CCl_4_-induced cirrhosis model mice (Fig. 1A), and serum biochemical tests and fibrosis accumulation were evaluated compared with the NS injection control group. Analyses of serum biochemical parameters revealed that serum levels of AST (NS group: 686 ± 101 IU/L, HMGB1 group: 108 ± 34 IU/L, *p* < 0.0001) and ALT (NS group: 488 ± 202 IU/L, HMGB1 group: 43 ± 9 IU/L, *p* = 0.0006) decreased significantly in the HMGB1 injection group compared with those in the NS control group (Fig. 1B). Although serum levels of T-Bil were not significantly altered, serum ALB levels were significantly increased (NS group 3.18 ± 0.17 g/dL, HMGB1 group 3.48 ± 0.12 g/dL, *p* = 0.0024) in the HMGB1 injection group compared with those in the NS control group (Fig. 1B). Evaluation of fibrosis demonstrated that the Sirius Red staining area (NS group: 0.63% ± 0.18%, HMGB1 group: 0.37% ± 0.12%, *p* = 0.0066; Fig. 1C and D) and hydroxyproline levels (NS group: 7.96 ± 0.97 nmol/mg, HMGB1 group: 4.56 ± 0.90 nmol/mg, *p* < 0.0001; Fig. 1E) were significantly decreased in the HMGB1 injection group compared with those in the NS control group. These results revealed that injection of HMGB1 peptide effectively attenuated liver damage, increased ALB production, and alleviated fibrosis.

### HMGB1 injection induced fibrolysis after 2 weeks

**Fig. 2:**
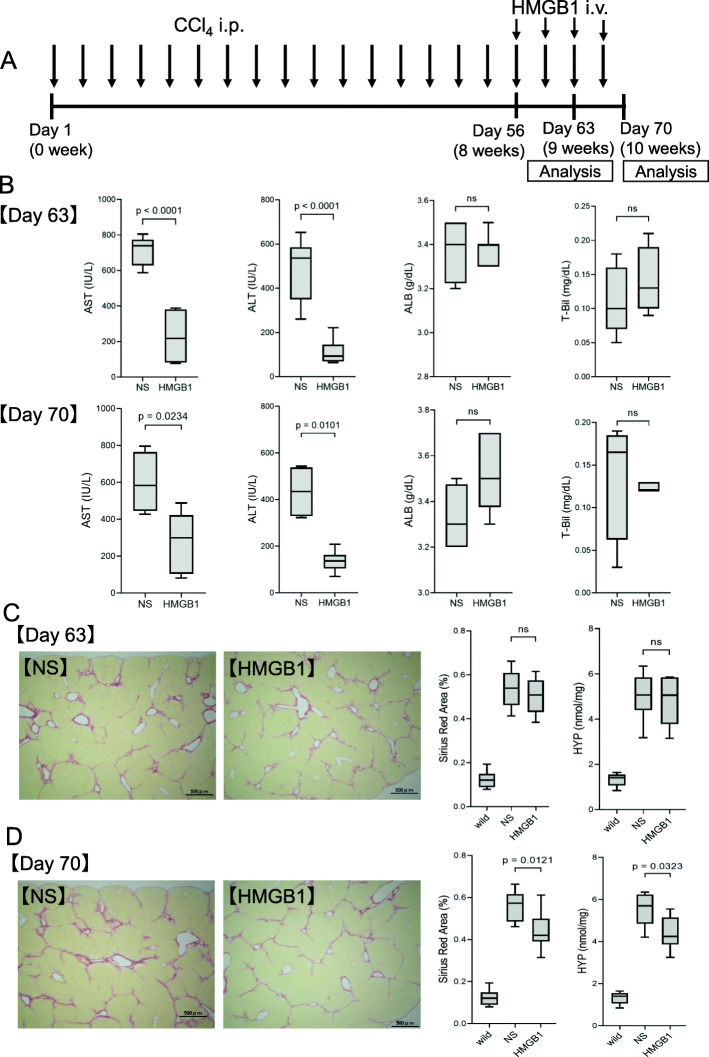
Therapeutic effects of HMGB1 peptide (1 or 2 weeks of treatment) in the CCl_4_-induced mouse cirrhosis model. **A** Schematic of this experiment. **B** Serum levels of AST, ALT, ALB, and T-Bil on days 63 (1 week of treatment) and 70 (2 weeks of treatment). **C** Sirius Red staining and quantification of hydroxyproline levels on day 63 (1 week of treatment) and **D** Sirius Red staining and quantification of hydroxyproline levels on day 70 (2 weeks of treatment). NS, normal saline, *n* = 8 in each group. Data are presented as means ± standard deviations. ns, not significant. wild, basic data of from undamaged normal liver at the same age

Next, we evaluated the timing of the effects of HMGB1 injection on attenuation of liver damage and regression of fibrosis following 1–2 weeks of HMGB1 peptide (5 μg/g) treatment with continuous CCl_4_ injection from 8 weeks after the beginning of CCl_4_ treatment (Fig. 2A). Serum levels of ALB and T-Bil did not change; however, serum AST (1 week, NS group: 713 ± 75 IU/L, HMGB1 group: 224 ± 132 IU/L, *p* < 0.0001; 2 weeks, NS group: 597 ± 115 IU/L, HMGB1 group: 280 ± 152 IU/L, *p* = 0.0234) and ALT (1 week, NS group: 492 ± 131 IU/L, HMGB1 group: 110 ± 52 IU/L, *p* < 0.0001; 2 weeks, NS group: 434 ± 100 IU/L, HMGB1 group: 136 ± 41 IU/L, *p* = 0.0101) levels were significantly decreased in the HMGB1 injection group compared with those in the control group (Fig. 2B). Moreover, after 1 week, the Sirius Red staining area and hydroxyproline level did not change significantly; however, after 2 weeks, significant decreases in Sirius Red staining area (NS group: 0.56% ± 0.07%, HMGB1 group: 0.44% ± 0.08%, *p* = 0.0121) and hydroxyproline levels (NS group: 5.53 ± 0.72 nmol/mg, HMGB1 group: 4.39 ± 0.73 nmol/mg, *p* = 0.0323) were observed in the HMGB1 injection group compared with those in the NS control group (Fig. 2C and D). In addition, both Sirius Red staining area and hydroxyproline level tended to decrease over time during continuous CCl_4_ treatment (Fig. 2C and D). These results revealed that reduction of liver damage occurred within 1 week after HMGB1 injection, and fibrosis regression, including fibrolysis, was observed within 2 weeks after HMGB1 injection.

### HMGB1 peptide does not cause a rapid reduction in liver damage in an acute liver damage model

**Fig. 3:**
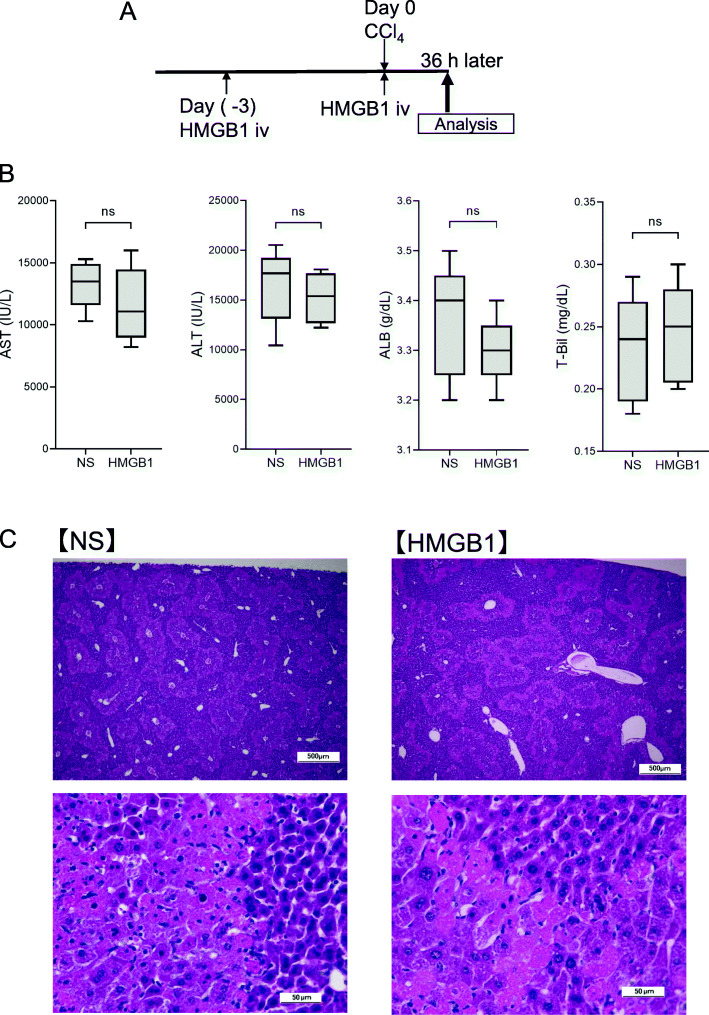
Therapeutic effects of HMGB1 in a one-time CCl_4_-induced liver damage model. **A** Schematic of this experiment. **B** Serum levels of AST, ALT, ALB, and T-Bil. **C** Hematoxylin and eosin staining (upper panels, scale bar = 500 μm; lower panels, scale bar: 50 μm). *n* = 8 in each group. Data are presented as means ± standard deviations. ns, not significant

Next, we evaluated the rapid attenuation of liver damage using a one-time CCl_4_ injection model in which HMGB1 peptide (5 μg/g) was injected two times (3 days prior to CCl_4_ injection and the same day of CCl_4_ injection; Fig. 3A). Serum markers and tissue damage were analyzed 36 h after the final CCl_4_ injection. AST, ALT, ALP, and T-Bil levels and tissue damage were not significantly altered (Fig. 3B and C), suggesting that at least 1 week of treatment may be required to detect the effects of HMGB1 peptide on attenuation of liver damage.

### HMGB1 peptide did not affect macrophages directly in vitro but altered macrophage polarization indirectly in vivo

**Fig. 4:**
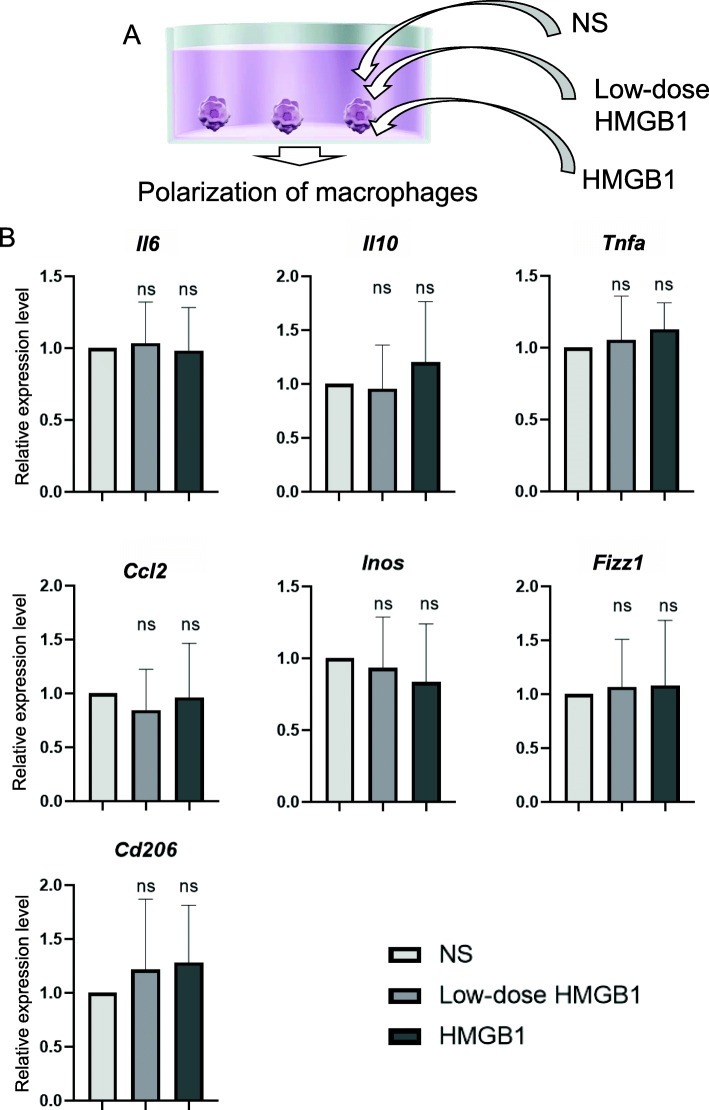
Effects of HMGB1 peptide against macrophages in vitro. **A** Schematic of this experiment. **B** After addition of normal saline (control), 0.01 mg (low dose) HMGB1 peptide, or 0.1 mg HMGB1 peptide to macrophage cultures, mRNA expression levels of *Il6*, *Il10*, *Tnfa*, *Ccl2*, *Inos*, *Fizz1*, and *Cd206* were analyzed (*n* = 5 for each experiment). Data are presented as means ± standard deviations. ns, not significant

Because macrophages have key roles in the regression of fibrosis, we next analyzed the direct effects of HMGB1 peptide against macrophages in vitro (Fig. 4A) and assessed changes in the mRNA expression of pro-inflammatory and anti-inflammatory markers. No significant changes in the expression levels of any markers were detected, suggesting that HMGB1 peptide did not exhibit direct effects in culture (Fig. 4B).

To analyze in-depth the immune cell profiles, a single-cell RNA-seq of CD45-positive cells was performed using liver tissues obtained 4 weeks after HMGB1 peptide injection. After conventional quality check and filtering, we recovered 6047 and 3668 cells from the HMGB1-treated liver (*n*=3) and control liver (*n*=3), respectively.

**Fig. 5:**
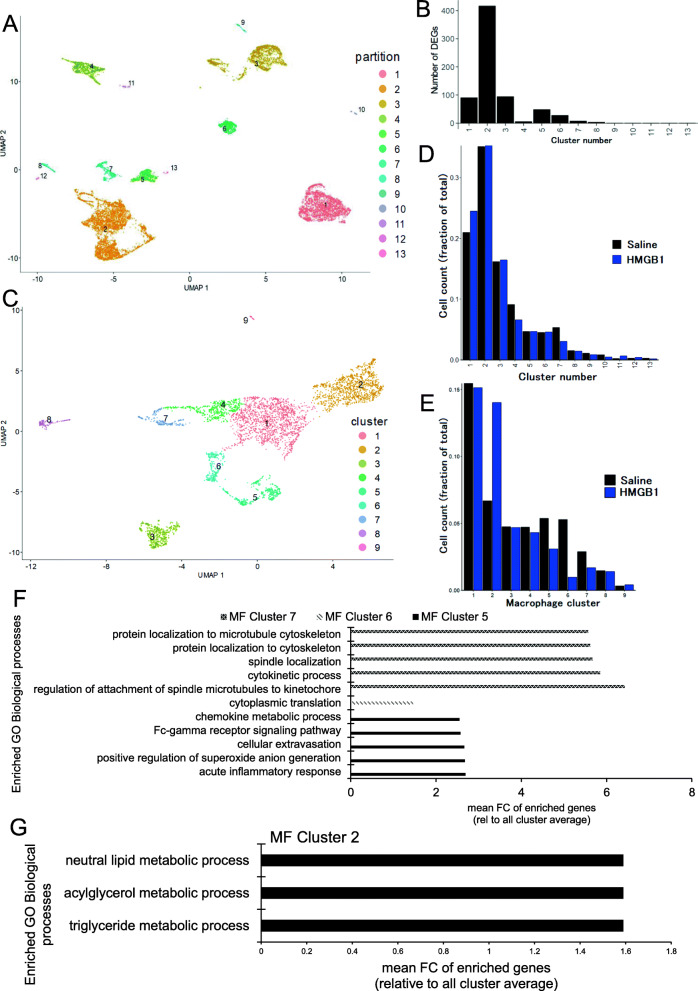
Effects of HMGB1 peptide analyzed by single-cell RNA-seq. **A** Summarized results are shown by Uniform Manifold Approximation and Projection (UMAP) plots. **B** The numbers of differentially expressed genes (*q* value < 0.05) in each partition in **A** are shown. **C** Reclustered UMAP of macrophage populations. **D** Frequencies of immune cells in each cluster in **A** in the normal saline (NS) control and HMGB1 peptide-treated groups. **E** The frequencies of macrophage subtypes in **C** in the normal saline (NS) control and HMGB1 peptide-treated groups. **F** Gene Ontology biological process functional analysis of subtypes 5, 6, and 7. **G** Gene Ontology analysis using genes that were solely expressed by macrophage subtype 2

As shown in Fig. 5A, thirteen partitions were identified. To identify the cell type responsive to the peptide treatment, we measured the number of differentially expressed genes (DEG) in each partition (Fig. 5B). The most responsive cell type was found to be included in partition 2, which is rich in macrophages. We thus investigated the phenotypical changes in the macrophage compartment.

**Fig. 6:**
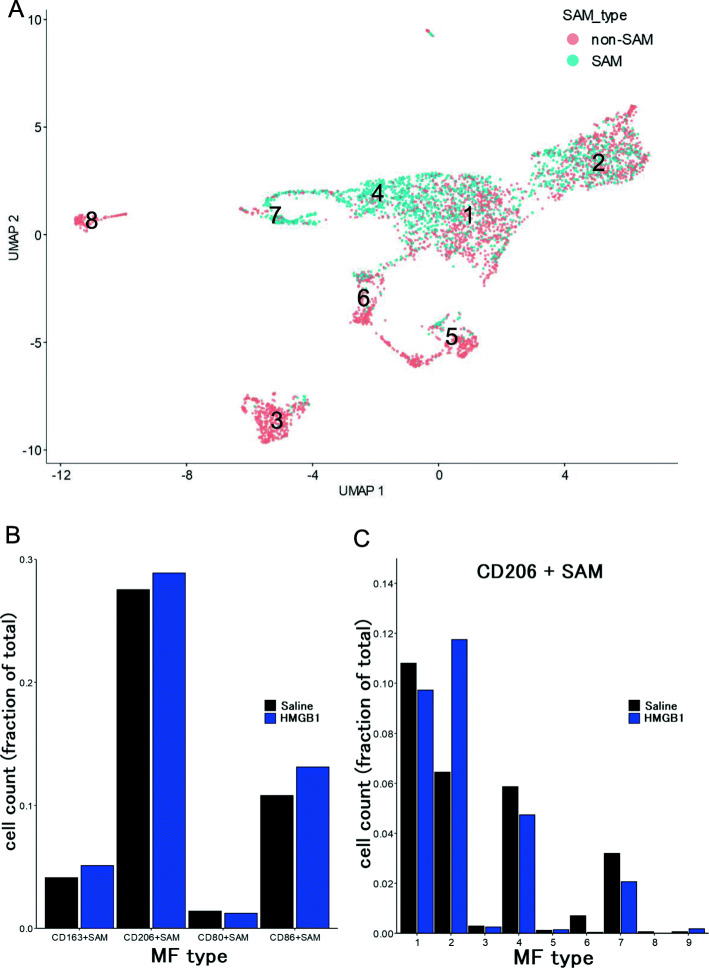
Detailed analysis of scar-associated macrophages (SAMs). **A** the distribution of SAMs and non-SAMs are shown. **B** the number of the cells of SAMs co-express inflammatory M1 markers (CD80 or CD86) or anti-inflammatory M2 markers (CD163 or CD206) are shown. **C** The frequency of CD206+SAMs in each macrophage subtype

To obtain macrophages for functional analyses, partitions positive for the macrophage markers *Emr1*, *Cd68*, and *C1qa* were reclustered to enrich for macrophages and deplete for non-macrophage cells. The resulted macrophage population forms nine clusters, which spread out on the UMAP (Fig. 5C), suggesting the distinct functionality of each cluster and thus represent macrophage subtypes. The distribution of some macrophage-related genes is shown in Fig. 6. We noticed that the peptide treatment did not impact the population size of macrophages, which is indicated by the unchanged population size of the macrophage-rich main cluster 2 (Fig. 5D). The treatment instead triggered phenotypical changes of macrophages, strongly suggested by the quantitative changes in the proportion of macrophage subtypes. Macrophage subtype 2, which is positive for *Clec4f* and is likely to represent Kupffer cells (KCs), increased by 110%, whereas subtypes 5, 6, and 7 decreased by 42%, 82%, and 42%, respectively (Fig. 5E). To elucidate the functions of these responsive subtypes, we aggregated genes with uneven expression distribution on the UMAP into modules by Monocle 3 and compared the aggregated gene expression levels among the different macrophage subtypes. Genes in modules showing deviation in expression from the average by more than 2 SDs were pooled and subjected to Gene Ontology analyses. Macrophage subtypes 5, 6, and 7 were associated with acute inflammatory responses, protein translation, and the cell cycle, respectively (Fig. 5F). Reduction of these macrophage subtypes indicates that HMGB1 treatment has suppressed the inflammatory responses in the liver. Gene Ontology analysis did not reveal functional enrichment for macrophage subtype 2, which was expanded by HMGB1. Nevertheless, this subtype preferentially contained *Cd163*+ and *Cd206+* cells (Supplemental Figure 1), which are both classical “M2” markers. Therefore, we speculated that some cells of macrophage subtype 2 may possess anti-inflammatory properties.

To gain further insights into how the peptide treatment affects the functions of the KC-like macrophage subtype 2, we focused on genes that showed greater than 2-fold changes in expression after HMGB1 treatment. These responsive genes were contributed differently by the nine macrophage subtypes, and we inspected the Gene Ontology of genes solely expressed by subtype 2. These treatment-responsive genes in subtype 2 seemed to be associated with lipid metabolism (Fig. 5G). As KCs are the major resident macrophages involved in regulating lipid metabolism in the healthy liver, the result proposes a recovered homeostatic macrophage function after peptide administration.

**Fig. 7:**
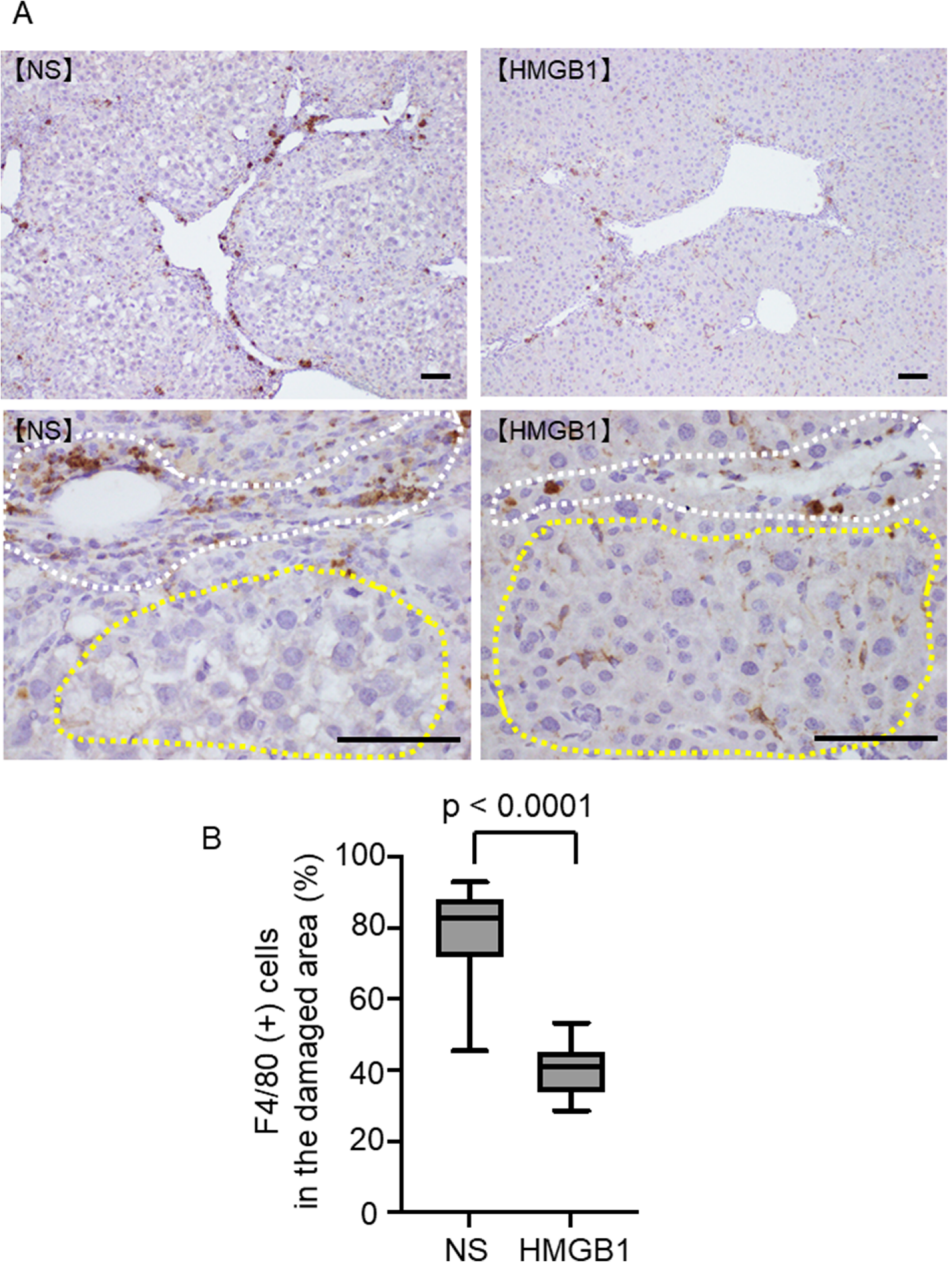
Immunostaining of liver tissues. **A** Immunostaining using F4/80 4 weeks after the treatment of NS or HMGB1 peptide is shown. **B** The frequency of F4/80+ cells in the damaged area is shown. White dotted circle, damaged area; yellow dotted area, parenchyma. Scale bar = 100 μm. *n* = 5 in each group. Data are presented as means ± standard deviations

Recently, Ramachandran et al. reported that the cells present in the fibrotic niche in the liver included the TREM2+ CD9+ subpopulation of scar-associated macrophages (SAMs) [20]. Thus, we examined whether SAM phenotypes had been altered by the treatment. SAMs are enriched within macrophage subtypes 1, 2, 4, and 7 (Fig. 6A). Amongst the SAMs, we noticed some cells being polarized and co-express inflammatory M1 markers (CD80 or CD86) or anti-inflammatory M2 markers (CD163 or CD206). In either treatment, CD206+ SAM was the most abundant polarized SAM phenotype (Fig. 6B). We hence analyzed the effect of HMGB1 treatment on the abundance of CD206+ SAM in each macrophage subcluster. We observed a specific increase in CD206+ SAM in KC-like subcluster 2 (Fig. 6C). In addition, we performed immunostaining for macrophages using anti-F4/80 antibodies and liver tissues 4 weeks after treatment. The results showed that at 4 weeks after treatment, number of F4/80+ macrophages in the damaged area were significantly decreased and were scattered to the parenchyma similar to the KC of the normal tissues (Fig. 7A and B). These results support that number of macrophages with anti-inflammatory KC-like subcluster 2 increased 4 weeks after the treatment.

## Discussion

In this study, we showed that HMGB1 peptide effectively attenuated liver damage and promoted the regression of fibrosis in a CCl_4_-induced cirrhosis model in mice. The effect of attenuating liver damage was not fast, but appeared within 1 week, whereas the effect of reducing fibrosis was slower and occurred after attenuating liver damage. Moreover, regression of fibrosis was caused by both a reduction in fibrogenesis and an increase in fibrolysis. Although macrophages were not directly affected by HMGB1 peptide, the most affected cells in the liver were macrophages. Notably, after HMGB1 peptide injection, the number of pro-inflammatory macrophages decreased, and that of macrophages with the KC phenotype and/or anti-inflammatory markers increased in the liver. These results were consistent with previous studies showing that macrophage or MSC plus macrophage therapy is effective for cirrhosis [6]. Although the approach to induce cirrhosis varied, our findings and these previous studies suggested that induction of anti-inflammatory macrophages may be essential for the treatment of cirrhosis.

HMGB1 is known to accelerate inflammation via the box B domain [11]. Gaskell et al. and Ge et al. provided a detailed review of how HMGB1 signaling participates in acute liver injury and chronic liver disease [21, 22]. Chen et al. reported that HMGB1 itself exacerbates CCl4-induced acute liver injury mouse model and blocking the HMGB1attenuate the damage of this model [23]. However, similar to this case, HMBG1 has also been reported to play key roles in tissue regeneration. Local administration of HMGB1 peptide promotes tissue regeneration in myocardial infarction or diabetic ulcers by attenuating inflammation and/or promoting angiogenesis [24, 25]. These effects are believed to be caused by box A of HMBG1. Moreover, in an acute liver injury model, transfer of the gene encoding HMGB1 box A could reduce liver damage [26]. Taken together, these results revealed that box A of HMGB1 was important for attenuating liver damage and reducing fibrosis.

In our study, we did not fully elucidate why macrophages were affected by HMGB1 peptide injection. Tamai et al. reported that HMGB1 can alter the motility of platelet-derived growth factor receptor α (PDGFRα)-positive MSCs and that these PDGFRα-positive MSCs in bone marrow can migrate to damaged skin. They also reported that both HMGB1 full-length protein and this partial HMGB1 peptide function similarly to mobilize PDGFRα-positive mesenchymal stem/progenitor cells from the bone marrow into circulation. On the other hand, this HMGB1 peptide is devoid of the TLR/RAGE-stimulating domains of HMGB1, making it noncapable of inflammatory activation [27, 28]. A previous study reported that recombinant box A prevented RAGE-dependent internalization of HMGB1, as well as HMGB1/LPS complexes in cultured macrophages [29]. However, Tamai et al. have reported using the same peptide used in our study that MSC-mobilization activity of HMGB1 was independent of RAGE stimulation that was proven by utilizing RAGE-depleting MSCs in vitro [28]. The potential effects of HMGB1 peptide on MSCs are quite interesting [30–32]. In our previous study, we showed that injection of cultured MSCs could alter the polarity of macrophages in vivo [6]. In this study, HMGB1 peptide did not affect macrophages directly, and it would be intriguing if HMGB1 peptide could affect macrophages through MSCs. Interestingly, Son et al. reported that C1q modulates the pro-inflammatory response induced by HMGB1 by collaborating with HMGB1 and inducing anti-inflammatory M2-like macrophages [33]. Thus, the specific mechanisms through which HMGB1 peptide can terminate inflammation through indirect effects on macrophages are still unclear.

In our study, single-cell RNA-seq enabled us to divide the macrophages into nine subtypes. Subtype 2 macrophages expressed KC markers and anti-inflammatory macrophage markers (*Cd206* and *Cd163*), and subtypes 5, 6, and 7 macrophages expressed potent pro-inflammatory markers. In addition, we elucidated that after HMGB1 peptide treatment, CD206+ SAM in KC-like subcluster 2 increased. Although the full multifunctionality of macrophages was not elucidated in our study, our approach provided important insights into cell and drug therapies for cirrhosis. However, we believe that single-cell RNA-seq is very helpful for the analysis of macrophage behavior after treatment.

HMGB1 peptide has been shown to have potent effects on tissue repair and has been already administered in various models in other fields. For example, Kido et al. reported that HMGB1 can prevent deterioration of cardiac function following injection into a hamster model of dilated cardiomyopathy [34]. Goto et al. also reported that this peptide promotes tissue repair in a rat model of myocardial infarction [15]. Moreover, HMGB1 peptide is being evaluated in clinical trials in Japan (S-005151, Redasemtide) for dystrophic epidermolysis bullosa (phase II) and acute ischemic stroke (phase II). Phase I studies have already confirmed safety. We set the dose of HMGB1 peptide as 5 mg/kg in this mouse study, because no severe adverse event was observed within 5 mg/kg doses of HMGB1 peptide in the phase I clinical trial (UMIN 000018252).

Thus, this peptide may have applications in the alleviation of chronic liver diseases with fibrosis accumulation. We will start phase II clinical studies in patients with chronic liver disease in the near future.

The HMGB1 peptide may have effects similar to those of cell therapies. Indeed, in our mouse cirrhosis model, we confirmed that HMGB1 peptide had similar effects as macrophage, MSC, and MSC and macrophage combination therapies. Cell therapy may induce several effects, such as induction of anti-inflammatory macrophages, inactivation of T cells, and induction of regulatory T cells [6, 35]. However, allogeneic macrophages are very difficult to use, and careful management of the culture process and quality is required. Although the peptide may have limited effects compared with cell therapies, culture is not needed, and quality control is easier than that with cells. Accordingly, this peptide may have applications in liver regeneration and reduction of fibrosis, and further studies are required to optimize the timing and dose of administration.

This study had some limitations. First, we employed only one animal model (CCl_4_-induced liver cirrhosis model). In the future, our concept should be evaluated in other models. Additionally, we could not confirm the mobilization of PDGFRα-positive MSCs in single-cell RNA-seq analyses. Dissociation of liver tissues while maintaining cell viability was challenging, and many parenchymal cells died during the single-cell RNA analyses. Thus, the establishment of modified liver dissociation methods may help us to resolve these problems. Performing the single-cell RNA-seq analysis at an early point after treatment might enable the elucidation of the mechanisms more precisely. This study did not dwell deep into the mechanistic aspects, and we could not analyze the direct effect of this peptide against hepatic stellate cells (HSCs). Recently, Zhang et al. reported that HSC-targeted lipid nanoparticles loaded with HMGB1-siRNA attenuated liver fibrosis and inflammation [36]. The difference between this study and ours is that while Zhang et al. used HMGB1 knockdown, we used the modified HMGB1 peptide lacking box B lesion for the treatment. Based on the results of our study, we speculate that decreasing the number of pro-inflammatory macrophages while increasing that of the anti-inflammatory macrophages with this peptide attenuated fibrogenesis and augmented fibrolysis.

However, our data clearly showed that this HMGB1 peptide is effective for liver cirrhosis model mouse, and up to now, there is no approved anti-fibrosis drug. Therefore, we believe that this result itself is very significant for the development of novel therapeutic strategies for chronic liver diseases in the clinic.

## Conclusions

Overall, in this study, we demonstrated the therapeutic effects of HMGB1 peptide in a mouse model of cirrhosis. Our findings provide a basis for future clinical studies of the potential therapeutic effects of HMGB1 peptide in patients with chronic liver diseases.

## Supplementary Information

**Additional file 1.** Supplementary material

## Data Availability

All data needed to evaluate the conclusions in the paper are present in the paper and/or the Supplementary Materials. Additional data related to this paper may be requested from the authors.

## Abbreviations

MSC: Mesenchymal stem cell
HMGB1: High mobility group box 1
CCl4: Carbon tetrachloride
PDGFRα: Platelet-derived growth factor receptor α
