# Supplementary material for: Synthesized HMGB1 peptide attenuates liver inflammation and suppresses fibrosis in mice
Source: Inflamm Regen. 2021 Sep 27;41:28. doi: 10.1186/s41232-021-00177-4 (PMC8474861; doi:10.1186/s41232-021-00177-4)

**Synthesized HMGB1 peptide attenuates liver inflammation and suppresses fibrosis in mice**

Shunsuke Nojiri^1^, Atsunori Tsuchiya^1^, Kazuki Natsui^1^, Suguru Takeuchi^1^, Takayuki Watanabe^1^, Yuichi Kojima^1^, Yusuke Watanabe^1^, Hiroteru Kamimura^1^, Masahiro Ogawa^1^, Satoko Motegi^1^, Takahiro Iwasawa^1^, Takeki Sato^1^, Masaru Kumagai^1^, Yui Ishii^1^, Tomomi Kitayama^2,3^, Yu-Tung Li^2^, Yuya Ouchi^2,3^, Takashi Shimbo^2,4^, Masaaki Takamura^1^, Katsuto Tamai^2^, Shuji Terai^1^

^1^Division of Gastroenterology and Hepatology, Graduate School of Medical and Dental Sciences, Niigata University, 1-757, Asahimachi-dori, Chuo-ku, Niigata 951-8510, Japan

^2^ Department of Stem Cell Therapy Science, Graduate School of Medicine, Osaka University, 2-2, Yamadaoka, Suita, Osaka 565-0871, Japan

^3^ StemRIM Inc., Saito Bio-Incubator 3F 7-7-15, Saito-Asagi, Ibaraki City, Osaka 567-0085, Japan

^4^ StemRIM Institute of Regeneration-Inducing Medicine, Osaka University, 2-8, Yamadaoka, Suita, Osaka 565-0871, Japan

**Supplementary Information**

**Supplemental Table 1.** List of primers used for real-time PCR.

| Primer | Catalog number | Species | Company |
| --- | --- | --- | --- |
| *Gapdh* | QT01658692 | Mouse | Qiagen |
| Pro-inflammatory macrophage factors | | | |
| *Il-6* | QT00098875 | Mouse | Qiagen |
| *Tnf-a* | QT00104006 | Mouse | Qiagen |
| *Inos* | QT01547980 | Mouse | Qiagen |
| *Ccl-2* | QT00167832 | Mouse | Qiagen |
| Anti-inflammatory macrophage factors | | | |
| *Il-10* | QT00106169 | Mouse | Qiagen |
| *Fizz-1* | QT00254359 | Mouse | Qiagen |
| *Cd206* | QT00103012 | Mouse | Qiagen |

**Supplemental Figure 1.** mRNA expression of markers in macrophage subtypes.
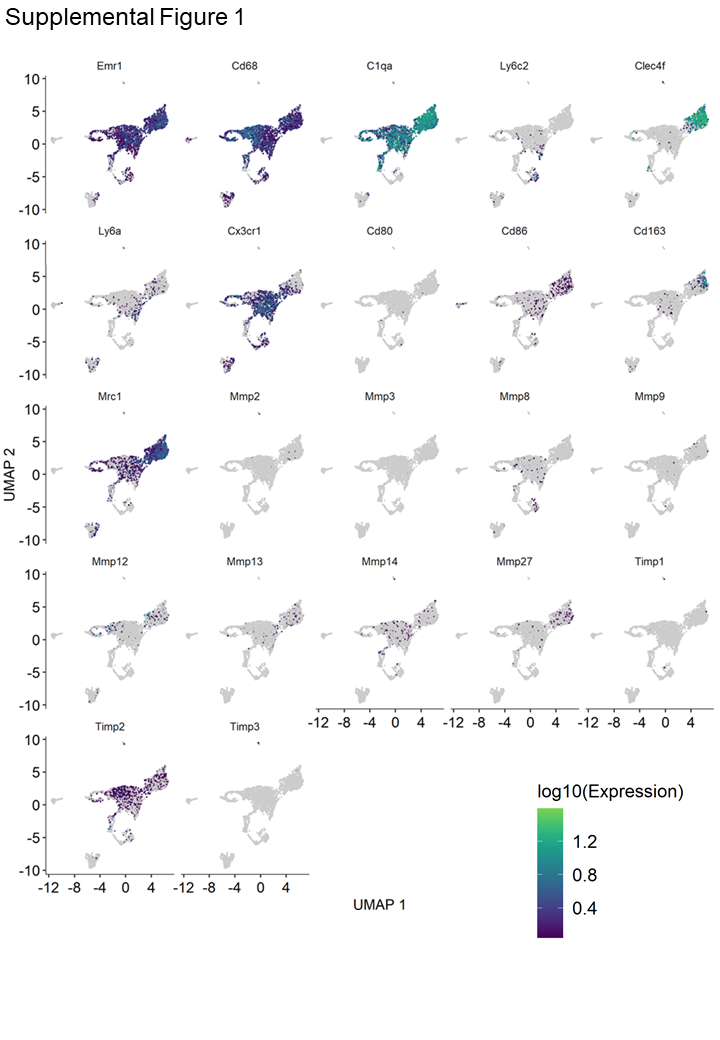

Supplement: Supplementary file 1 — Additional file 1. Supplementary material [file 41232_2021_177_MOESM1_ESM.docx]
